# 3-[(*E*)-3,7-Dimethyl­octa-2,6-dien­yl]-5-methyl-*N*-nitro-1,3,5-oxadiazinan-4-imine

**DOI:** 10.1107/S160053680801492X

**Published:** 2008-05-24

**Authors:** Tie-Niu Kang, Li Zhang, Yun Ling, Xin-Ling Yang

**Affiliations:** a Department of Applied Chemistry, China Agricultural University, Beijing 100094, People’s Republic of China

## Abstract

The title compound, C_14_H_24_N_4_O_3_, was synthesized by the reaction of geranyl and 3-methyl-4-nitro­imino-1,3,5-oxadiazinane. In the crystal structure, mol­ecules are assembled by weak inter­molecular C—H⋯O hydrogen bonds. The nitryl and the long carbon chain are located on the same side of the C=N bond due to the two weak intra­molecular C—H⋯N hydrogen bonds; the configuration of the oxadiazinane is *Z*.

## Related literature

For background literature, see: Bowers *et al.* (1972 ▶). For related literature, see: Yang *et al.* (2004 ▶); Van Oosten *et al.* (1990 ▶).
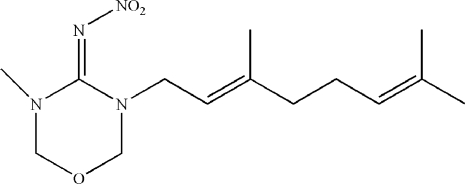


## Experimental

### 

#### Crystal data

C_14_H_24_N_4_O_3_
*M* *_r_* = 296.37
Monoclinic, 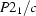

*a* = 7.9318 (16) Å
*b* = 6.6423 (13) Å
*c* = 31.191 (7) Å
β = 99.55 (3)°
*V* = 1620.5 (6) Å^3^
*Z* = 4
Mo *K*α radiation
μ = 0.09 mm^−1^
*T* = 293 (2) K
0.60 × 0.30 × 0.08 mm

#### Data collection

Rigaku R-AXIS RAPID IP diffractometer
Absorption correction: multi-scan (*ABSCOR*; Higashi, 1995 ▶) *T*_min_ = 0.943, *T*_max_ = 0.993
7692 measured reflections
2825 independent reflections
1306 reflections with *I* > 2σ(*I*)
*R*_int_ = 0.0508

#### Refinement

*R*[*F* ^2^ > 2σ(*F* ^2^)] = 0.047
*wR*(*F* ^2^) = 0.152
*S* = 0.84
2825 reflections
194 parameters
H-atom parameters constrained
Δρ_max_ = 0.28 e Å^−3^
Δρ_min_ = −0.30 e Å^−3^

### 

Data collection: *RAPID-AUTO* (Rigaku, 2000 ▶); cell refinement: *RAPID-AUTO*; data reduction: *CrystalStructure* (Rigaku/MSC, 2000 ▶); program(s) used to solve structure: *SHELXS97* (Sheldrick, 2008 ▶); program(s) used to refine structure: *SHELXL97* (Sheldrick, 2008 ▶); molecular graphics: *SHELXTL* (Sheldrick, 2008 ▶); software used to prepare material for publication: *SHELXL97*.

## Supplementary Material

Crystal structure: contains datablocks I, global. DOI: 10.1107/S160053680801492X/rk2083sup1.cif

Structure factors: contains datablocks I. DOI: 10.1107/S160053680801492X/rk2083Isup2.hkl

Additional supplementary materials:  crystallographic information; 3D view; checkCIF report

## Figures and Tables

**Table 1:** Hydrogen-bond geometry (Å, °)

*D*—H⋯*A*	*D*—H	H⋯*A*	*D*⋯*A*	*D*—H⋯*A*
C2—H2*B*⋯O1^i^	0.97	2.48	3.257 (4)	136
C3—H3*A*⋯N2^ii^	0.97	2.43	3.336 (4)	155
C3—H3*B*⋯O1^iii^	0.97	2.38	3.264 (4)	151
C5—H5*B*⋯N1	0.97	2.53	3.117 (4)	119
C5—H5*B*⋯N2	0.97	2.55	2.960 (4)	105
C13—H13*C*⋯O2^iv^	0.96	2.59	3.425 (5)	145

Symmetry codes: (i) 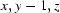; (ii) 
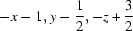
; (iii) 
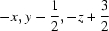
; (iv) 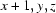.

**Fig. 1.**
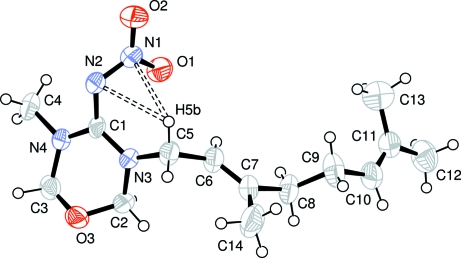
The molecular structure of I with the atom numbering scheme. The displacement ellipsoids are drawn at 50% probability level. H atoms are presented as a small spheres of arbitrary radius. Intramolecular hydrogen bonds are shown as dashed lines.

**Fig. 2.**
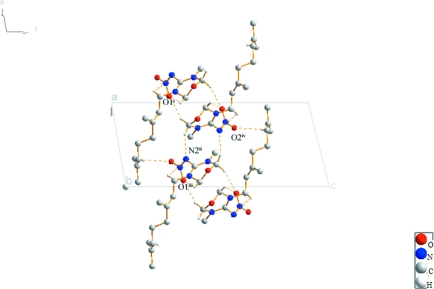
The crystal packing of I. Hydrogen bonds are shown as dashed lines. Symmetry codes: (i) x, y-1, z; (ii) -x-1, y-1/2, -z+3/2; (iii) -x, y-1/2, -z+3/2; (iv) x+1, y, z.

## supplementary crystallographic information

### Comment

*E-b*-farnesene (*EBF*), the primary component of aphides alarm pheromone, not only stimulate the movement of aphid (Bowers *et al.*, 1972), but also possess the acute activity to many economically aphid species at a dose of 100 ng/aphid (Van Oosten *et al.*, 1990). However, *EBF* is limited in field application due to its high volatility, readily air oxidation and degradation under field conditions. In order to improve its chemical stability and biological efficacy, the pharmacophore of neonicotinoids was introduced to substitute the conjugated double bond of *EBF* (Yang *et al.*, 2004). The title compound (I), in which 3-methyl-5-(*E*)-3,7-dimethylocta-2,6-dienyl connect to *N*-nitro-1,3,5-oxadiazinan-4-imine instead of the conjugated double bond, was synthesized as *EBF* analogue with potent insecticidal activity. To study the further structure-activity relationship, we reported here its molecular and crystal structure. The molecular structure showed *Z*-isomer by the interaction forces of weak intramolecular C5–H5b···N1 and C5–H5b···N2 hydrogen bonds (Fig. 1). The compound was assembled by four weak intermolecular hydrogen bonds (Fig. 2 and Table 1).

### Experimental

To a solution of 3-methyl-*N*-nitro-1,3,5-oxadiazinan-4-imine (1.60 g, 10.0 mmol) dissolved in anhydrous acetonitrile (15 ml), geranyl (1.89 g, 10.1 mmol) was added. Then the reaction solution was slowly heated to reflux for 7h. After removing the solvent, the residue was purified by column chromatography on silica gel (200-300 mesh) with petroleum ether/ethylacetate (2.5:1*v/v*) as eluent to obtain the title compound I. Then, 50 mg I was dissolved in 20 ml me thanol. The solution was kept at room temperature for 20 d by natural evaporation to give colorless single crystals of I, suitable for X-Ray analysis.

^1^H NMR (CDCl_3_, 300 MHz) 1.60 (s, 3H, CH_3_–C═C), 1.68 [s, 6H, (CH_3_)_2_C═C], 2.07~2.10 (t, J = 5.27 Hz, 4H, -CH_2_–CH_2_-), 3.05 (s, 3H, N–CH_3_), 4.11 (d, J = 7.26, 2H,-CH_2_–N), 4.09 (s, 2H, N–CH_2_–O), 4.12 (s, 2H, O–CH_2_–N), 5.03~5.18 (m, 2H, 2CH═C); Calc. for C_14_H_24_N_4_O_3_: C 56.74, H 8.16, N 18.90; found C 56.69, H 8.19, N 18.80.

### Refinement

The H atoms were fixed geometrically and allowed to ride on their parent atoms, with C–H = 0.93-0.97 Å, and with *U_iso_*(H) = *1.2U_eq_* for (C_aromatic_ and C_methylene_) or *U*_iso_(H) = 1.5*U*_eq_(C_methyl_). The intensities of equivalent reflections were merged (*R_int_* = 0.000).

### Crystal data

**Table:**

C_14_H_24_N_4_O_3_	*F*_000_ = 640
*M**_r_* = 296.37	*D*_x_ = 1.215 Mg m^−^^3^
Monoclinic, *P*2_1_/*c*	Mo *K*α radiation λ = 0.71073 Å
Hall symbol: -P 2ybc	Cell parameters from 7693 reflections
*a* = 7.9318 (16) Å	θ = 2.6–25.0º
*b* = 6.6423 (13) Å	µ = 0.09 mm^−^^1^
*c* = 31.191 (7) Å	*T* = 293 (2) K
β = 99.55 (3)º	Block, colourless
*V* = 1620.5 (6) Å^3^	0.60 × 0.30 × 0.08 mm
*Z* = 4	

### Data collection

**Table:**

Rigaku R-AXIS RAPID IP diffractometer	2825 independent reflections
Radiation source: Fine-focus sealed tube	1306 reflections with *I* > 2σ(*I*)
Monochromator: Graphite	*R*_int_ = 0.051
Detector resolution: 10.00 pixels mm^-1^	θ_max_ = 25.0º
*T* = 293(2) K	θ_min_ = 1.3º
ω scans	*h* = 0→9
Absorption correction: multi-scan(ABSCOR; Higashi, 1995)	*k* = −7→0
*T*_min_ = 0.943, *T*_max_ = 0.993	*l* = −37→35
2825 measured reflections	

### Refinement

**Table:**

Refinement on *F*^2^	Secondary atom site location: difference Fourier map
Least-squares matrix: full	Hydrogen site location: inferred from neighbouring sites
*R*[*F*^2^ > 2σ(*F*^2^)] = 0.047	H-atom parameters constrained
*wR*(*F*^2^) = 0.153	*w* = 1/[σ^2^(*F*_o_^2^) + (0.0843*P*)^2^] where *P* = (*F*_o_^2^ + 2*F*_c_^2^)/3
*S* = 0.84	(Δ/σ)_max_ = 0.014
2825 reflections	Δρ_max_ = 0.28 e Å^−^^3^
194 parameters	Δρ_min_ = −0.30 e Å^−^^3^
Primary atom site location: structure-invariant direct methods	Extinction correction: None

### Special details

**Table:**

Geometry. All s.u.'s (except the s.u. in the dihedral angle between two l.s. planes) are estimated using the full covariance matrix. The cell s.u.'s are taken into account individually in the estimation of s.u.'s in distances, angles and torsion angles; correlations between s.u.'s in cell parameters are only used when they are defined by crystal symmetry. An approximate (isotropic) treatment of cell s.u.'s is used for estimating s.u.'s involving l.s. planes.
Refinement. Refinement of *F*^2^ against ALL reflections. The weighted *R*-factor *wR* and goodness of fit *S* are based on *F*^2^, conventional *R*-factors *R* are based on *F*, with *F* set to zero for negative *F*^2^. The threshold expression of *F*^2^ > σ(*F*^2^) is used only for calculating *R*-factors(gt) *etc*. and is not relevant to the choice of reflections for refinement. *R*-factors based on *F*^2^ are statistically about twice as large as those based on *F*, and *R*-factors based on ALL data will be even larger.

### Fractional atomic coordinates and isotropic or equivalent isotropic displacement parameters (Å^2^)

**Table:**

	*x*	*y*	*z*	*U*_iso_*/*U*_eq_	
O1	−0.0768 (3)	0.9179 (3)	0.82543 (8)	0.0604 (7)	
O2	−0.2995 (3)	1.0170 (4)	0.85187 (8)	0.0670 (7)	
O3	−0.1951 (2)	0.2689 (3)	0.74730 (7)	0.0512 (6)	
N1	−0.2308 (3)	0.9002 (4)	0.82899 (8)	0.0459 (7)	
N2	−0.3292 (3)	0.7617 (4)	0.80698 (8)	0.0453 (7)	
N3	−0.1290 (3)	0.4939 (4)	0.80512 (8)	0.0414 (6)	
N4	−0.3001 (3)	0.6021 (4)	0.74324 (8)	0.0392 (6)	
C1	−0.2455 (3)	0.6227 (4)	0.78538 (9)	0.0362 (7)	
C2	−0.0594 (4)	0.3511 (5)	0.77744 (10)	0.0472 (8)	
H2a	0.0212	0.4183	0.7621	0.057*	
H2b	0.0004	0.2445	0.7950	0.057*	
C3	−0.2672 (4)	0.4183 (5)	0.71928 (10)	0.0501 (9)	
H3a	−0.3739	0.3699	0.7027	0.060*	
H3b	−0.1907	0.4502	0.6990	0.060*	
C4	−0.4042 (4)	0.7564 (5)	0.71773 (10)	0.0560 (9)	
H4a	−0.3882	0.7482	0.6879	0.084*	
H4b	−0.3700	0.8871	0.7291	0.084*	
H4c	−0.5225	0.7348	0.7195	0.084*	
C5	−0.0734 (3)	0.4762 (5)	0.85230 (9)	0.0439 (8)	
H5a	−0.1016	0.3433	0.8619	0.053*	
H5b	−0.1332	0.5747	0.8672	0.053*	
C6	0.1153 (4)	0.5098 (5)	0.86376 (10)	0.0474 (8)	
H6	0.1567	0.6275	0.8532	0.057*	
C7	0.2293 (4)	0.3932 (5)	0.88694 (10)	0.0499 (8)	
C8	0.4169 (4)	0.4518 (6)	0.89472 (10)	0.0608 (10)	
H8a	0.4835	0.3403	0.8864	0.073*	
H8b	0.4328	0.5654	0.8763	0.073*	
C9	0.4840 (4)	0.5072 (7)	0.94162 (11)	0.0740 (11)	
H9a	0.4240	0.6254	0.9494	0.089*	
H9b	0.4615	0.3976	0.9604	0.089*	
C10	0.6736 (4)	0.5496 (6)	0.94863 (11)	0.0624 (10)	
H10	0.7444	0.4383	0.9483	0.075*	
C11	0.7505 (4)	0.7241 (6)	0.95516 (10)	0.0597 (9)	
C12	0.9427 (5)	0.7390 (6)	0.96099 (13)	0.0838 (13)	
H12a	0.9742	0.8405	0.9419	0.126*	
H12b	0.9892	0.6117	0.9542	0.126*	
H12c	0.9870	0.7744	0.9906	0.126*	
C13	0.6634 (6)	0.9221 (6)	0.95783 (15)	0.0974 (14)	
H13a	0.7091	0.9870	0.9848	0.146*	
H13b	0.5430	0.9007	0.9565	0.146*	
H13c	0.6824	1.0058	0.9340	0.146*	
C14	0.1868 (5)	0.2002 (6)	0.90720 (13)	0.0861 (14)	
H14a	0.2656	0.0975	0.9016	0.129*	
H14b	0.0725	0.1605	0.8950	0.129*	
H14c	0.1950	0.2183	0.9380	0.129*	

### Atomic displacement parameters (Å^2^)

**Table:**

	*U*^11^	*U*^22^	*U*^33^	*U*^12^	*U*^13^	*U*^23^
O1	0.0374 (12)	0.0485 (14)	0.0951 (18)	−0.0067 (11)	0.0103 (12)	−0.0033 (13)
O2	0.0714 (16)	0.0645 (16)	0.0645 (15)	0.0175 (13)	0.0094 (12)	−0.0191 (13)
O3	0.0433 (12)	0.0422 (12)	0.0677 (14)	−0.0059 (11)	0.0082 (11)	−0.0098 (12)
N1	0.0461 (17)	0.0434 (16)	0.0465 (16)	0.0077 (14)	0.0025 (13)	0.0017 (14)
N2	0.0296 (13)	0.0510 (16)	0.0534 (16)	0.0058 (13)	0.0011 (12)	−0.0096 (14)
N3	0.0336 (13)	0.0400 (14)	0.0478 (15)	0.0059 (12)	−0.0012 (11)	−0.0038 (13)
N4	0.0296 (12)	0.0432 (15)	0.0435 (15)	−0.0001 (11)	0.0027 (11)	−0.0017 (13)
C1	0.0183 (14)	0.0412 (18)	0.0479 (19)	−0.0034 (13)	0.0021 (13)	0.0002 (15)
C2	0.0379 (18)	0.0395 (18)	0.062 (2)	0.0052 (15)	0.0016 (15)	−0.0042 (17)
C3	0.0330 (17)	0.061 (2)	0.056 (2)	−0.0053 (16)	0.0070 (15)	−0.0113 (19)
C4	0.0399 (17)	0.074 (2)	0.0498 (19)	0.0089 (18)	−0.0048 (15)	0.0077 (18)
C5	0.0368 (16)	0.0465 (19)	0.0465 (18)	−0.0003 (15)	0.0014 (14)	0.0047 (16)
C6	0.0393 (17)	0.0513 (19)	0.0485 (18)	−0.0036 (16)	−0.0022 (14)	0.0069 (17)
C7	0.0458 (18)	0.056 (2)	0.0440 (18)	0.0006 (17)	−0.0053 (14)	−0.0021 (17)
C8	0.0424 (18)	0.082 (3)	0.053 (2)	0.0028 (18)	−0.0071 (16)	0.0019 (19)
C9	0.053 (2)	0.108 (3)	0.056 (2)	−0.015 (2)	−0.0043 (17)	−0.010 (2)
C10	0.046 (2)	0.072 (3)	0.065 (2)	−0.0023 (19)	−0.0049 (17)	−0.008 (2)
C11	0.057 (2)	0.066 (3)	0.053 (2)	0.000 (2)	−0.0002 (17)	−0.0038 (19)
C12	0.059 (2)	0.097 (3)	0.091 (3)	−0.022 (2)	0.002 (2)	−0.011 (3)
C13	0.100 (3)	0.084 (3)	0.105 (4)	0.010 (3)	0.008 (3)	−0.007 (3)
C14	0.078 (3)	0.067 (3)	0.097 (3)	−0.005 (2)	−0.032 (2)	0.027 (2)

### Geometric parameters (Å, °)

**Table:**

O1—N1	1.251 (3)	C6—H6	0.9300
O2—N1	1.240 (3)	C7—C14	1.493 (5)
O3—C3	1.382 (3)	C7—C8	1.518 (4)
O3—C2	1.416 (3)	C8—C9	1.517 (4)
N1—N2	1.323 (3)	C8—H8a	0.9700
N2—C1	1.376 (3)	C8—H8b	0.9700
N3—C1	1.333 (3)	C9—C10	1.510 (4)
N3—C2	1.452 (4)	C9—H9a	0.9700
N3—C5	1.469 (4)	C9—H9b	0.9700
N4—C1	1.321 (3)	C10—C11	1.310 (5)
N4—C4	1.465 (4)	C10—H10	0.9300
N4—C3	1.477 (4)	C11—C13	1.494 (5)
C2—H2a	0.9700	C11—C12	1.508 (5)
C2—H2b	0.9700	C12—H12a	0.9600
C3—H3a	0.9700	C12—H12b	0.9600
C3—H3b	0.9700	C12—H12c	0.9600
C4—H4a	0.9600	C13—H13a	0.9600
C4—H4b	0.9600	C13—H13b	0.9600
C4—H4c	0.9600	C13—H13c	0.9600
C5—C6	1.496 (4)	C14—H14a	0.9600
C5—H5a	0.9700	C14—H14b	0.9600
C5—H5b	0.9700	C14—H14c	0.9600
C6—C7	1.313 (4)		
			
C3—O3—C2	109.4 (2)	C5—C6—H6	116.1
O2—N1—O1	121.4 (3)	C6—C7—C14	123.8 (3)
O2—N1—N2	117.2 (3)	C6—C7—C8	120.3 (3)
O1—N1—N2	121.3 (3)	C14—C7—C8	115.9 (3)
N1—N2—C1	115.5 (2)	C9—C8—C7	113.2 (3)
C1—N3—C2	116.6 (2)	C9—C8—H8a	108.9
C1—N3—C5	125.7 (2)	C7—C8—H8a	108.9
C2—N3—C5	117.6 (2)	C9—C8—H8b	108.9
C1—N4—C4	122.0 (2)	C7—C8—H8b	108.9
C1—N4—C3	122.2 (2)	H8a—C8—H8b	107.8
C4—N4—C3	115.7 (2)	C10—C9—C8	111.5 (3)
N4—C1—N3	118.7 (3)	C10—C9—H9a	109.3
N4—C1—N2	116.8 (2)	C8—C9—H9a	109.3
N3—C1—N2	123.9 (3)	C10—C9—H9b	109.3
O3—C2—N3	108.9 (2)	C8—C9—H9b	109.3
O3—C2—H2a	109.9	H9a—C9—H9b	108.0
N3—C2—H2a	109.9	C11—C10—C9	127.9 (4)
O3—C2—H2b	109.9	C11—C10—H10	116.0
N3—C2—H2b	109.9	C9—C10—H10	116.0
H2a—C2—H2b	108.3	C10—C11—C13	125.4 (3)
O3—C3—N4	111.3 (2)	C10—C11—C12	120.8 (3)
O3—C3—H3a	109.4	C13—C11—C12	113.7 (3)
N4—C3—H3a	109.4	C11—C12—H12a	109.5
O3—C3—H3b	109.4	C11—C12—H12b	109.5
N4—C3—H3b	109.4	H12a—C12—H12b	109.5
H3a—C3—H3b	108.0	C11—C12—H12c	109.5
N4—C4—H4a	109.5	H12a—C12—H12c	109.5
N4—C4—H4b	109.5	H12b—C12—H12c	109.5
H4a—C4—H4b	109.5	C11—C13—H13a	109.5
N4—C4—H4c	109.5	C11—C13—H13b	109.5
H4a—C4—H4c	109.5	H13a—C13—H13b	109.5
H4b—C4—H4c	109.5	C11—C13—H13c	109.5
N3—C5—C6	110.5 (2)	H13a—C13—H13c	109.5
N3—C5—H5a	109.6	H13b—C13—H13c	109.5
C6—C5—H5a	109.6	C7—C14—H14a	109.5
N3—C5—H5b	109.6	C7—C14—H14b	109.5
C6—C5—H5b	109.6	H14a—C14—H14b	109.5
H5a—C5—H5b	108.1	C7—C14—H14c	109.5
C7—C6—C5	127.9 (3)	H14a—C14—H14c	109.5
C7—C6—H6	116.1	H14b—C14—H14c	109.5

### Hydrogen-bond geometry (Å, °)

**Table:**

*D*—H···*A*	*D*—H	H···*A*	*D*···*A*	*D*—H···*A*
C2—H2B···O1^i^	0.97	2.48	3.257 (4)	136
C3—H3A···N2^ii^	0.97	2.43	3.336 (4)	155
C3—H3B···O1^iii^	0.97	2.38	3.264 (4)	151
C5—H5B···N1	0.97	2.53	3.117 (4)	119
C5—H5B···N2	0.97	2.55	2.960 (4)	105
C13—H13C···O2^iv^	0.96	2.59	3.425 (5)	145

Symmetry codes: (i) *x*, *y*−1, *z*; (ii) −*x*−1, *y*−1/2, −*z*+3/2; (iii) −*x*, *y*−1/2, −*z*+3/2; (iv) *x*+1, *y*, *z*.
